# Association of NT-proBNP With Clinical Outcomes in Patients Undergoing Transcatheter Tricuspid Valve Intervention

**DOI:** 10.1016/j.jacadv.2026.102921

**Published:** 2026-06-17

**Authors:** Athanasios Feidakis, Jan Althoff, Thorsten Gietzen, Laura Marx, Caroline Hasse, Jennifer von Stein, Maria I. Körber, Stephan Baldus, Roman Pfister, Christos Iliadis

**Affiliations:** Faculty of Medicine and University Hospital Cologne, Department III of Internal Medicine, University of Cologne, Cologne, Germany

**Keywords:** biomarkers, NT-proBNP, right heart failure, risk stratification, transcatheter tricuspid valve intervention, tricuspid regurgitation

## Abstract

**Background:**

N-terminal pro–B-type natriuretic peptide (NT-proBNP) is a well-established biomarker for risk stratification in heart failure. However, its prognostic value and early postprocedural dynamics in patients undergoing transcatheter tricuspid valve intervention (TTVI) remain incompletely defined.

**Objectives:**

The objective of the study was to investigate the association of baseline NT-proBNP levels and early postinterventional NT-proBNP changes with long-term all-cause mortality and symptomatic improvement after TTVI.

**Methods:**

We analyzed a cohort of patients with severe tricuspid regurgitation undergoing TTVI. Patients were stratified according to baseline NT-proBNP tertiles. Changes in NT-proBNP between baseline and 4-week follow-up were assessed, and their association with long-term all-cause mortality was evaluated using Kaplan-Meier analysis and Cox regression. NYHA functional class improvement (≥1 class) at 4 weeks was analyzed as a secondary endpoint using logistic regression.

**Results:**

Higher baseline NT-proBNP levels were independently associated with increased long-term all-cause mortality. Early NT-proBNP changes provided additional prognostic information: patients with increasing NT-proBNP levels at 4 weeks showed markedly worse survival, irrespective of procedural success. In contrast, baseline NT-proBNP was not associated with symptomatic improvement according to NYHA functional class at 4-week follow-up. Patients with increasing NT-proBNP levels exhibited larger right atrial volumes and higher right atrial pressures at baseline, suggesting more advanced right-sided remodeling.

**Conclusions:**

In patients undergoing TTVI, baseline NT-proBNP levels and early postprocedural increases in NT-proBNP identify individuals at high risk for long-term mortality but do not predict symptomatic improvement. NT-proBNP reflects residual myocardial stress rather than procedural success and may aid in patient selection and early postinterventional surveillance.

Tricuspid regurgitation (TR), historically considered an “innocent bystander” to left-sided heart disease, has gained increased clinical attention due to mounting evidence of its significant impact on morbidity and mortality.^1^ Due to a rapidly aging population and increased awareness of the adverse consequences of untreated TR, its clinical burden is steadily increasing.^2^ Traditionally, TR was often managed conservatively due to high surgical risk and limited specific treatment options. However, transcatheter tricuspid valve interventions (TTVIs) have emerged as a viable therapeutic alternative for symptomatic patients deemed inoperable or at high surgical risk, offering a less invasive approach with promising early outcomes.^3^

Despite these advances, identifying reliable prognostic markers in this unique patient population remains a challenge. Established surgical or transcatheter risk scores lack the use of biomarkers. Natriuretic peptides are well-established biomarkers used for risk-stratification and prediction of both hospitalization and mortality in patients with heart failure (HF).^4^ Although N-terminal pro–B-type natriuretic peptide (NT-proBNP) has been predominantly applied in this context, its prognostic relevance in valvular heart disease (VHD) has gained substantial attention in recent years. Among conservatively treated patients with moderate or severe VHD, including TR, higher age- and sex-adjusted NT-proBNP ratios have been independently associated with increased mortality, demonstrating a consistent and robust prognostic value across multiple VHD subtypes.^5^ Beyond medically managed cohorts, natriuretic peptides also carry prognostic information in patients undergoing invasive treatment for severe VHD: lower baseline NT-proBNP levels have been associated with more favorable long-term outcomes in aortic stenosis patients undergoing transcatheter aortic valve replacement (TAVR),^6^ as well as in individuals with severe mitral regurgitation treated with transcatheter edge-to-edge repair (TEER).^7^

The present study aimed to evaluate the prognostic relevance of NT-proBNP levels, both at baseline and 4 weeks after transcatheter tricuspid valve repair, and to determine their association with long-term mortality in patients with severe, symptomatic TR treated with TTVI.

## Methods

### Study design and population

This retrospective observational study included all consecutive patients with symptomatic TR, who underwent percutaneous TTVI at our single tertiary care center between January 2018 and December 2023. Patients with previous severe concomitant left-sided valvular disease (aortic stenosis/regurgitation or mitral regurgitation) were eligible only if the lesion had been adequately treated prior to the index procedure (eg, prior TAVR, surgical aortic valve replacement or mitral repair/replacement/TEER, with no more than mild residual dysfunction). All patients underwent a standardized diagnostic work-up including a transesophageal echocardiography. Patients with increased transtricuspid gradient in echocardiography underwent right heart catheterization at baseline. The decision to pursue transcatheter intervention instead of surgery, along with the selection of the specific device, was made by an interdisciplinary heart team. All patients were deemed at high surgical risk.

Patients with unavailable baseline NT-proBNP values or with technically unsuccessful device implantation, defined as failure to successfully deliver and deploy the intended device(s) in tricuspid valve position and retrieve the delivery system, were excluded. Because of their distinct pathophysiological mechanisms and hemodynamic effects, patients who underwent heterotopic caval valve implantation using stent-grafts were also excluded.

Patients were scheduled for standardized follow-up at 4 weeks and 12 months after the procedure, which included blood sampling, transthoracic echocardiography, and clinical examination.

Clinical, laboratory, and echocardiographic data were obtained retrospectively from the hospital’s electronic medical records. The study protocol was approved by the institutional ethics committee and conducted in accordance with the Declaration of Helsinki.

### Outcome definitions

The primary endpoint was all-cause mortality at 24 months. NYHA functional class improvement was assessed as a secondary endpoint and defined as an improvement of at least 1 NYHA functional class compared with baseline at 4-week follow-up.

Exploratory endpoints included the association of NT-proBNP levels at 4 weeks and absolute change in NT-proBNP between baseline and 4-week follow-up (Δ NT-proBNP) with all-cause mortality. Patients who died before 4-week NT-proBNP assessment were not included in these exploratory analyses.

### Event definitions

Death events were ascertained through review of hospital records, outpatient documentation, telephone follow-up, and correspondence from referring physicians. Patients were censored at the last available clinical contact if no event had occurred.

Major bleeding events were defined according to the International Society on Thrombosis and Haemostasis criteria, including fatal bleeding, symptomatic bleeding in a critical area or organ, or bleeding causing a hemoglobin drop of ≥2 g/dL or requiring transfusion of ≥2 U of blood.^8^ Thromboembolic events were defined as a composite of ischemic stroke, transient ischemic attack, and myocardial infarction (MI) occurring during the index hospitalization. Daily loop diuretic dose dosage was determined as furosemide equivalent (furosemide: torasemide, 2: 1) in mg.

### Causal TR classification and TTVI procedure

TR etiology was classified as primary, secondary, or related to cardiac implantable electronic device according to the Tricuspid Valve Academic Research Consortium (TVARC) consensus definitions.^9^

Secondary TR was further classified according to underlying etiology. This included: 1) prior left VHD (aortic or mitral valvular disease previously treated); 2) left ventricular (LV) HF (HF with reduced ejection fraction, HF with mildly reduced ejection fraction, or HF with preserved ejection fraction); 3) pulmonary hypertension (pulmonary artery [PA] systolic pressure ≥50 mm Hg); and 4) isolated TR (atrial fibrillation without fulfilling other etiology).

The procedures included edge-to-edge repair using the PASCAL Ace system (Edwards Lifesciences) and direct annuloplasty with Cardioband (Edwards Lifesciences). Intraprocedural success was defined according to TVARC criteria.^9^

### Echocardiographic evaluation

Transthoracic echocardiographic assessments were conducted at baseline, discharge, and follow-up visits by experienced sonographers using standardized acquisition protocols in accordance with current American Society of Echocardiography/European Association of Cardiovascular Imaging guidelines.^10^ The severity of TR was graded according to the widely adopted 5-grade classification system proposed by Hahn et al^11^

### Statistical analysis

Continuous variables are presented as median (Q1-Q3), whereas categorical variables were summarized as counts and percentages. Group comparisons were performed using the chi-square test for categorical variables, and the Kruskal-Wallis or Mann-Whitney U tests for continuous variables, as appropriate. Paired data were analyzed with the Wilcoxon signed-rank or McNemar test. Missing data were handled using complete-case analysis for each individual statistical test and regression model (pairwise exclusion).

Survival time-to-event analyses were conducted using the Kaplan-Meier estimates and compared by log-rank testing. Univariable and multivariable Cox proportional hazards regression models were employed to assess associations between NT-proBNP variables and all-cause mortality. Selection of covariates for multivariable adjustment was primarily based on clinical relevance and potential confounding of the relationship between NT-proBNP and mortality, and was secondarily informed by univariable regression analyses. Covariate selection was additionally guided by a directed acyclic graph representing the presumed confounding structure between NT-proBNP and all-cause mortality (Supplemental Figure 1). To avoid multicollinearity in the multivariable Cox regression models, variable selection was further refined using the variance inflation factor. Variables with a variance inflation factor >5 were considered collinear and excluded from the final model. A 2-sided *P* value <0.05 was considered statistically significant. The proportional hazards assumption was assessed using Schoenfeld residuals. The association between baseline NT-proBNP and NYHA functional class improvement at each time point was evaluated using logistic regression models. Analyses were performed using IBM SPSS Statistics (version 23; IBM Corp) and R (version 4.4.2; R Foundation for Statistical Computing).

### NT-proBNP

Due to their markedly skewed distribution, logarithmic base-10 transformation of NT-proBNP values (log_10_[NT-proBNP]) both at baseline and 4 weeks was applied for all regression analyses. NT-proBNP at baseline was analyzed both as a continuous variable and after stratification by tertiles. Restricted cubic spline analysis was first performed using baseline log_10_ (NT-proBNP) as a continuous variable to explore the relationship between NT-proBNP and all-cause mortality and to assess for potential nonlinearity. As spline analysis demonstrated a significant association with mortality (overall *P* = 0.004) without evidence of substantial nonlinearity (*P* for nonlinearity = 0.279), tertiles were subsequently chosen to provide balanced group sizes and to facilitate descriptive comparisons and Kaplan-Meier visualization across increasing NT-proBNP levels.

The change in NT-proBNP between baseline and 4-week follow-up (Δ NT-proBNP) was examined as a continuous measure, as well as a binary variable indicating whether NT-proBNP at 4-week follow-up increased or decreased compared with baseline. To account for both the direction and magnitude of change, the absolute difference was transformed using a signed logarithmic approach, calculated as the sign of the raw difference multiplied by the logarithm of the absolute difference plus 1:

$$\text{Signed}\log_{10}\left(\Delta \text{NT}-\text{proBNP}\right)=\text{sign}\;\left(\Delta \;\text{NT}-\text{proBNP}\right)\times \log_{10\;}\left(\left|\Delta \;\text{NT}-\text{proBNP}\right|+1\right)$$

This transformation preserves the information about the direction of change (increase or decrease) while reducing skewness and stabilizing variance.

Analyses involving 4-week NT-proBNP values and Δ NT-proBNP were performed as landmark-type analyses among patients who survived until the 4-week follow-up visit and had available NT-proBNP reassessment. To evaluate potential selection bias related to missing 4-week NT-proBNP measurements, baseline characteristics of patients with and without available 4-week NT-proBNP values were additionally compared.

NT-proBNP concentrations were measured using an electrochemiluminescence immunoassay on the Roche Diagnostics platform.

## Results

### Baseline characteristics

A total of 339 patients consecutively treated with TTVI were initially screened. After applying the exclusion criteria, 293 patients were included in the current analysis (16 TricValve patients, 10 technically unsuccessful implantations, and 20 patients with missing baseline NT-proBNP values were excluded). The median age was 80 (76-83) years and 69% were females. Most patients (84%) presented with advanced symptoms, classified as NYHA functional class III or IV at baseline. The median NT-proBNP concentration was 2,016 ng/L (1,265-3,833) in the overall cohort. As outlined in the Methods section, patients were stratified into tertiles based on their baseline NT-proBNP values. The cutoff values used to define the tertiles were: low tertile: 47 to 1,519 ng/L; intermediate tertile: 1,519 to 2,996 ng/L; and high tertile: 3,009 to 48,803 ng/L. Due to tied NT-proBNP values at the tertile cutoff, minimal overlap in the observed ranges occurred despite unique patient assignment by rank-based tertile categorization. In respective tertiles, the median NT-proBNP was 915 ng/L in the low, 2,016 ng/L in the intermediate, and 4,828 ng/L in the high tertile.

Although patients in the intermediate and high NT-proBNP groups tended to be older, had a higher burden of comorbidities, such as atrial fibrillation and chronic kidney disease, and required increased loop diuretic dose compared with the low tertile, no significant differences were observed in symptomatic status across tertiles, as reflected by comparable NYHA functional class distribution (Table 1).

**Table 1:** Baseline Clinical Characteristics Across NT-proBNP Tertiles

	Total (N = 293)	Low Tertile (n = 98)	Intermediate Tertile (n = 98)	High Tertile (n = 97)	*P*^26^ Value
Age, y	80 (76-83)	80 (75-82)	81 (76-84)	81 (78-85)	0.029
Female	202 (69)	69 (70)	74 (76)	59 (61)	0.080
BMI, kg/m^2^	24.69 (22.07-28.30)	25.41 (22.98-28.91)	25.54 (22.92-29)	23.73 (21.64-26.42)	0.019
EuroSCORE II, %	4.35 (2.77-7.24)	3.23 (2.06-4.88)	4.49 (2.79-7.632)	4.89 (3.63-8.43)	<0.001
TRI-SCORE, points	5 (3-6)	4 (2-6)	5 (3-6)	6 (4-7)	<0.001
TR-Etiology					0.30
Primary	2 (0.7)	0 (0)	1 (1)	1 (1)	
Functional	263 (90)	90 (92)	89 (91)	84 (87)	
CIED-related	19 (6.5)	3 (3.1)	6 (6.1)	10 (10)	
Mixed	9 (3.1)	5 (5.1)	2 (2)	2 (2.1)	
NYHA functional class					0.40
Class-I	4 (1.4)	3 (3.1)	0 (0)	1 (1)	
Class-II	43 (15)	17 (17)	16 (16)	10 (10)	
Class-III	214 (73)	68 (69)	70 (71)	76 (78)	
Class-IV	32 (11)	10 (10)	12 (12)	10 (10)	
HF-hospitalization Last 12 Mo	150 (51)	36 (37)	56 (57)	58 (60)	0.002
Left heart failure phenotypes					0.004
No left HF	17 (5.8)	10 (10)	2 (2)	5 (5.2)	
HFrEF	15 (5.1)	1 (1)	3 (3.1)	11 (11)	
HFmrEF	34 (12)	8 (8.2)	13 (13)	13 (13)	
HFpEF	227 (77)	79 (81)	80 (82)	68 (70)	
Hypertension	234 (80)	76 (78)	82 (84)	76 (78)	0.50
Diabetes	63 (22)	16 (16)	23 (23)	24 (25)	0.30
Stroke/TIA	40 (14)	12 (12)	12 (12)	16 (16)	0.60
Atrial fibrillation	271 (92)	85 (87)	96 (98)	90 (93)	0.012
COPD	41 (14)	10 (10)	11 (11)	20 (21)	0.070
CKD	150 (51)	49 (50)	41 (42)	60 (62)	0.019
Terminal CKD on dialysis	6 (2)	3 (3.1)	1 (1)	2 (2.1)	0.60
CAD	118 (40)	34 (35)	37 (38)	47 (48)	0.12
Prior MI	17 (5.8)	5 (5.1)	2 (2)	10 (10)	0.051
Prior transcatheter left-sided valve intervention	53 (18)	17 (17)	13 (13)	23 (24)	0.20
Prior cardiac Surgery	64 (22)	26 (27)	20 (20)	18 (19)	0.40
CIED	67 (23)	13 (13)	26 (27)	28 (29)	0.020
Thiazides	52 (18)	12 (12)	16 (16)	24 (25)	0.067
MRA	134 (46)	44 (45)	43 (44)	47 (48)	0.80
Beta blockers	254 (87)	77 (79)	85 (87)	92 (95)	0.004
ARNi	16 (5.5)	3 (3.1)	5 (5.1)	8 (8.2)	0.30
RAS-blockers	130 (44)	38 (39)	52 (53)	40 (41)	0.10
SGLT2-inhibitors	63 (21.5)	17 (19)	21 (25)	25 (30)	0.20
Loop diuretic dose, mg	40 (20-80)	20 (10-60)	40 (20-80)	40 (20-80)	<0.001

Values are median (IQR) or n (%).

ARNi = angiotensin receptor neprilysin inhibitor; BMI = body mass index; CAD = coronary artery disease; CIED = cardiac implanted electronic device; CKD = chronic kidney disease; COPD = chronic obstructive pulmonary disease; HF = heart failure; HFpEF = heart failure with preserved ejection fraction; HFmrEF = heart failure with mildly reduced ejection fraction; HFrEF = heart failure with reduced ejection fraction; MI = myocardial infarction; MRA = mineralocorticoid receptor antagonist; NT-proBNP = N-terminal pro–B-type natriuretic peptide; RAS = renin angiotensin system; SGLT2 = sodium-glucose co-transporter 2; TIA = transient ischemic attack; TR = tricuspid regurgitation.

In echocardiography, patients in the higher NT-proBNP tertiles exhibited a slightly worse LV ejection fraction and larger left atrial volume index. Patients in the intermediate tertile showed a modestly higher proportion of massive (IV°) and torrential (V°) TR. In addition, higher NT-proBNP tertiles were associated with worse right ventricular (RV) function, reflected by lower tricuspid annular plane systolic excursion and fractional area change values, as well as higher PA systolic pressure. In contrast, right heart structural parameters including right atrial (RA) volume index (RAVi) and RV basal diameter were comparable across groups (Table 2).

**Table 2:** Baseline Laboratory Findings and Echocardiographic Characteristics Across NT-proBNP Tertiles

	Total (N = 293)	Low Tertile (n = 98)	Intermediate Tertile (n = 98)	High Tertile (n = 97)	*P* Value
Laboratory findings					
Albumin, g/L	42 (39-44)	44 (41-45)	41 (39-43)	41 (38-43)	<0.001
Hemoglobin, g/dL	12.1 (10.65-13.20')	12.55 (11.1-13.6)	12.1 (10.5-13.1)	11.55 (10.3-12.8)	0.002
Anemia	159 (54)	40 (41)	53 (54)	66 (69)	<0.001
Creatinine, mg/dL	1.26 (0.95-1.68)	0.99 (0.85-1.29)	1.26 (0.99-1.58)	1.63 (1.22-2.13)	<0.001
eGFR, mL/min/1.73 m^2^	41 (31-53)	51 (38-62)	40.5 (33-51)	32 (23-42)	<0.001
Bilirubin, mg/dL	0.7 (0.5-1)	0.6 (0.5-1)	0.7 (0.5-1)	0.8 (0.5-1.1)	0.20
AST, U/L	29 (24-35)	29 (25-33)	28 (24-35)	30 (23-39)	0.80
ALT, U/L	18 (14-24)	20 (15-26)	18 (13-22)	17 (14-23)	0.032
NT-proBNP, ng/L	2,016 (1,265-3,833)	915 (579-1,265)	2,016 (1,787-2,486)	4,828 (3,876-6,980)	<0.001
Echocardiographic characteristics					
LVEDd, mm (n = 283)	46 (42-51)	46 (41-50)	46 (42-51)	47 (42-53)	0.3
LVEF, % (n = 261)	55 (52-61.7)	58 (54-62.5)	55 (52-60)	54.9 (49-61)	0.003
LAVi, mL/m^2^ (n = 261)	55 (42.8-71)	49.1 (37-65.9)	56 (44.3-69.3)	58.2 (46.4-81.9)	0.014
RAVi, mL/m^2^ (n = 224)	72 (54.2-101.9)	67.1 (53.4-93)	72 (55.4-97)	75.7 (55-110.8)	0.40
RV Basal Diameter, mm (n = 279)	45 (40.8-50.1)	45 (40.1-49.6)	44 (40-50)	46 (42-52)	0.30
TAPSE, mm (n = 282)	18 (14-21)	19 (16-22)	18 (14-20)	16 (13-19)	<0.001
FAC, % (n = 261)	35.5 (30-41)	38 (34-42.5)	35.4 (29.5-41.1)	32.5 (26.5-39.1)	<0.001
PASP, mm Hg (n = 271)	42.9 (34.9-54)	40 (35.15-49.5)	39.7 (33-52.75)	50 (35-58)	0.020
TR-grade at baseline					0.038
III°	173 (59)	65 (66)	47 (48)	61 (63)	
IV°	75 (26)	23 (23)	28 (29)	24 (25)	
V°	45 (15)	10 (10)	23 (23)	12 (12)	

Values are median (IQR) or n (%). For each variable, the value in parentheses (n = …) denotes the number of patients with available data. Variables without an “n = …” annotation had complete data with no missing observations.

ALT = alanine aminotransferase; AST = aspartate aminotransferase; eGFR = estimated glomerular filtration rate; FAC = fractional area change; LAVi = left atrial volume index; LVEDd = left ventricular end-diastolic diameter; LVEF = left ventricular ejection fraction; PASP = pulmonary artery systolic pressure; RAVi = right atrial volume index; RV = right ventricle; TAPSE = tricuspid annular plane systolic excursion; other abbreviations as in Table 1.

To ensure that exclusion of patients with technically unsuccessful device implantation did not introduce selection bias toward a less advanced disease stage, we compared the baseline characteristics of the excluded patients with those of the final study cohort. No statistically significant differences were observed, including for baseline NT-proBNP levels (Supplemental Table 1).

### Procedural and clinical outcomes

Edge-to-edge repair was performed in 66% of patients, whereas direct annuloplasty was used in 34%. An overall intraprocedural success rate of 70%, as defined by the TVARC criteria, was achieved. At discharge, 68% of patients exhibited less than severe TR (grade ≤ II). The median length of hospital stay was 6 days (5-9).

Apart from a slightly longer hospitalization duration in the intermediate and high NT-proBNP groups, no significant differences were observed across tertiles in terms of intraprocedural success, acute kidney injury, major bleeding complications, or thromboembolic events during hospital stay. Procedural and clinical outcomes according to NT-proBNP tertiles are summarized in Table 3.

**Table 3:** Procedural and Clinical Outcomes Across NT-proBNP Tertiles

	Total (N = 293)	Low Tertile (n = 98)	Intermediate Tertile (n = 98)	High Tertile (n = 97)	*P* Value
Type of intervention:					0.30
TEER	194 (66)	70 (71)	60 (61)	64 (66)	
CardioBand	99 (34)	28 (29)	38 (39)	33 (34)	
Intraprocedural Success (TVARC)	205 (70)	76 (78)	68 (69)	61 (63)	0.082
TR-grade at discharge					0.12
No TR	4 (1.4)	3 (3.1)	1 (1)	0 (0)	
I°	89 (30)	36 (37)	27 (28)	26 (27)	
II°	108 (37)	36 (37)	41 (42)	31 (32)	
III°	74 (25)	17 (17)	23 (23)	34 (35)	
IV°	15 (5.1)	4 (4.1)	5 (5.1)	6 (6.2)	
V°	3 (1)	2 (2)	1 (1)	0 (0)	
TV-inflow at discharge,mm Hg (n = 197)	1.8 (1.3-2.4)	2 (1.5-1.7)	1.7 (1.2-2.3)	1.6 (1.3-2.4)	0.10
Length of index hospitalization (days)	6 (5-9)	6 (4-7)	7 (5-10)	6 (5-12)	0.021
AKI during hospital stay	20 (6.8)	5 (5.1)	7 (7.1)	8 (8.2)	0.70
In-hospital major bleeding	19 (6.4)	4 (4.1)	8 (8.2)	7 (7.1)	0.50
In-hospital thromboembolic events	4 (1.4)	1 (1)	0 (0)	3 (3.1)	0.20
In-hospital mortality	5 (1.7)	0 (0)	1 (1)	4 (4.1)	0.069
NYHA functional class at 4 wk (n = 255)					0.20
Class-I	35 (14)	14 (15)	12 (13)	9 (12)	
Class-II	132 (52)	56 (61)	43 (48)	33 (45)	
Class-III	79 (31)	19 (21)	30 (34)	30 (41)	
Class-IV	9 (3.5)	3 (3.3)	4 (4.5)	2 (2.7)	
NYHA functional class at 1 y (n = 149)					0.30
Class-I	26 (17)	14 (26)	7 (13)	5 (13)	
Class-II	75 (50)	23 (43)	34 (61)	18 (46)	
Class-III	45 (30)	16 (30)	14 (25)	15 (38)	
Class-IV	3 (2)	1 (1.9)	1 (1.8)	1 (2.6)	

Values are median (IQR) or n (%). For each variable, the value in parentheses (n = …) denotes the number of patients with available data. Variables without an “n = …” annotation had complete data with no missing observations.

Thromboembolic events: composite of ischemic stroke, TIA, or MI. Major bleeding: ISTH definition-fatal bleeding, critical-organ bleeding, hemoglobin drop ≥2 g/dL, or transfusion ≥2 U.

AKI = Acute Kidney Injury; TEER = Transcatheter Edge-to-Edge Repair; TV = tricuspid valve; TVARC = Tricuspid Valve Academic Research Consortium; other abbreviations as in Table 1.

### Survival analysis

**Figure 1:**
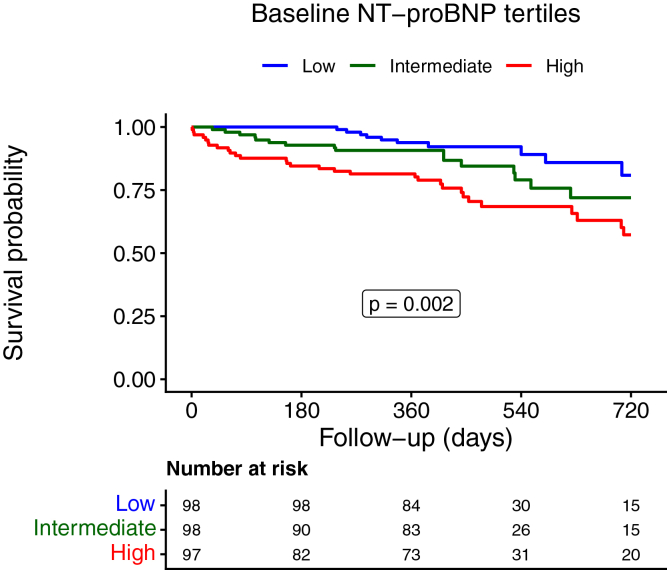
**Kaplan-Meier Curves for All-Cause Mortality by NT-proBNP Tertiles After Transcatheter Tricuspid Valve Intervention** This figure illustrates all-cause mortality stratified by baseline NT-proBNP tertiles in patients undergoing TTVI. Survival probabilities were estimated using the Kaplan-Meier method and compared using the log-rank test. Increasing baseline NT-proBNP tertiles were associated with a stepwise increase in mortality, with the highest tertile exhibiting the poorest long-term survival. NT-proBNP = N-terminal pro-B-type natriuretic peptide.

During a median follow-up of 410 days (IQR: 368-593), a total of 63 patients (21.5%) died. Of these, 33 deaths occurred within the first year, corresponding to 11.3% of the overall cohort and 52.4% of all deaths. By 2 years, 54 patients (18.4%) had reached the primary endpoint of all-cause mortality. Kaplan-Meier analysis demonstrated a significant stepwise increase in all-cause mortality across NT-proBNP tertiles, with patients in the highest tertile exhibiting the worst survival outcomes (log-rank *P* = 0.002) (Figure 1).

When assessed as a continuous variable, univariable Cox regression revealed that higher NT-proBNP levels at baseline were associated with an increased hazard for all-cause mortality (HR, 3.17; 95% CI, 1.82-5.50; *P* < 0.001). This association remained independently significant after multivariable adjustment for clinical and echocardiographic covariates (HR, 2.48; 95% CI, 1.25-4.91; *P* = 0.009).

In the adjusted model, additional independent predictors of all-cause mortality included a higher baseline dose of loop diuretics (per 20 mg furosemide-equivalent increase; HR, 1.044; 95% CI, 1.006-1.084; *P* = 0.022). Conversely, intraprocedural success was associated with a significantly lower risk of mortality (HR: 0.40; 95% CI: 0.23-0.70; *P* = 0.001).

Prior MI was associated with higher baseline NT-proBNP tertiles but was not significantly associated with mortality in univariable Cox analysis and was therefore not retained in the final multivariable model. In a sensitivity analysis excluding patients with prior MI, baseline log_10_(NT-proBNP) remained independently associated with all-cause mortality (HR: 2.364; 95% CI: 1.195-4.678; *P* = 0.013).

Device-specific sensitivity analyses demonstrated that baseline log_10_(NT-proBNP) was significantly associated with all-cause mortality in both TEER patients (HR: 2.94; 95% CI: 1.44-5.98; *P* = 0.003) and CardioBand patients (HR: 4.10; 95% CI: 1.65-10.16; *P* = 0.002). In an unadjusted interaction Cox model including baseline log_10_(NT-proBNP), device type, and their interaction, no significant interaction between device type and baseline NT-proBNP was observed (*P* for interaction = 0.433), suggesting that the prognostic value of NT-proBNP was not device specific.

The results of the univariable and multivariable Cox regression analyses for all-cause mortality are summarized in Table 4.

**Table 4:** Univariable and Multivariable Cox Regression Analyses for All-Cause Mortality

	Univariable Analysis			Multivariable Analysis		
	HR	95% CI	*P* Value	HR	95% CI	*P* Value
Log_10_ (NT-proBNP) at baseline	3.165	1.822-5.496	<0.001	2.479	1.252-4.909	0.009
Age	0.986	0.955-1.019	0.416			
Female	0.585	0.354-0.967	0.037	0.902	0.512-1.589	0.721
Prior MI	0.740	0.232-2.360	0.611			
Intraprocedural success (TVARC)	0.365	0.222-0.598	<0.001	0.404	0.234-0.699	0.001
Daily dose of loop-diuretics at baseline (per 20 mg furosemide-equivalent increase)	1.079	1.046-1.113	<0.001	1.044	1.006-1.084	0.022
Anemia at baseline	1.927	1.124-3.302	0.017	1.080	0.601-1.943	0.797
eGFR at baseline	0.972	0.956-0.988	<0.001	0.984	0.996-1.064	0.090
LVEDd at baseline	1.036	0.997-1.077	0.071			
LVEF at baseline	0.980	0.955-1.004	0.107			
RV basal diameter at baseline	1.055	1.024-1.088	<0.001	1.030	0.996-1.064	0.082

Patients included in the multivariable analysis: 279

Anemia was defined according to WHO criteria as hemoglobin <13 g/dL in men and <12 g/dL in women.

HRs for log_10_(NT-proBNP) variables are expressed per 1-U increase.

Abbreviations as in Table 1, Table 2, and 3.

**Figure 2:**
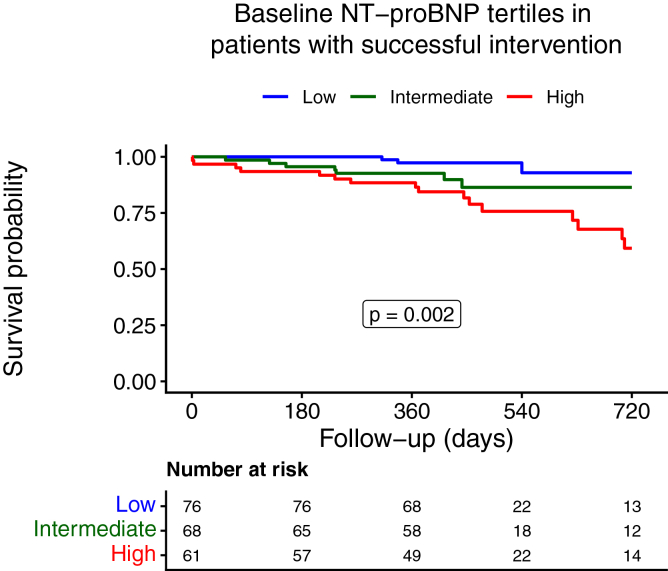
**Kaplan-Meier Curves for All-Cause Mortality by baseline NT-proBNP Tertiles Among Patients With Intraprocedural Success After Transcatheter Tricuspid Valve Intervention** This figure illustrates all-cause mortality stratified by baseline NT-proBNP tertiles in patients with intraprocedural success undergoing TTVI. Survival probabilities were estimated using the Kaplan-Meier method and compared using the log-rank test. Increasing baseline NT-proBNP tertiles remained associated with a stepwise increase in mortality despite successful intervention, with the highest tertile exhibiting the poorest long-term survival. Abbreviation as in Figure 1.

Baseline NT-proBNP retained its prognostic value even after technically successful TTVI, with its tertiles remaining significantly associated with long-term mortality among patients with intraprocedural success. Kaplan-Meier analysis demonstrated progressively worse survival across increasing tertiles despite successful intervention (log-rank *P* = 0.002) (Figure 2).

### Secondary endpoint

Baseline log_10_(NT-proBNP) was not significantly associated with NYHA functional class improvement at 4 weeks (OR: 0.78; 95% CI: 0.43-1.41; *P* = 0.417).

### Outcomes after 4-week follow-up and prognostic impact of early NT-proBNP change

At the 4-week follow-up, 255 patients (87%) underwent repeat clinical, laboratory, and echocardiographic assessment. Seven patients (2.4%) died within the first 30 days following the index procedure, including both in-hospital deaths and those occurring after discharge, whereas 31 patients (10.6%) were lost to follow-up. A substantial symptomatic improvement was observed, with 66% of the patients being classified in NYHA functional class I and II (compared to only 16.4% preprocedural). Overall, 57.7% of patients experienced an improvement of at least 1 NYHA functional class at 4-week follow-up. NT-proBNP measurements at the 4-week visit were available for 210 patients. Among those, the median NT-proBNP concentration at 4 weeks was 1,906.5 ng/L (1,133.5-3,265.2). Baseline characteristics of patients with and without available 4-week NT-proBNP measurements were compared. No clinically meaningful differences were observed between groups (Supplemental Table 2).

To assess the prognostic relevance of short-term NT-proBNP changes after TTVI, patients were dichotomized based on whether their NT-proBNP levels increased or decreased compared to baseline. Alternative stratification thresholds for 4-week NT-proBNP change, including percentage-based cutoffs, are presented in the Supplemental Appendix. Table 5 summarizes the baseline clinical, laboratory, and echocardiographic characteristics of the 2 groups. Overall, there were no differences in clinical, laboratory, and echocardiographic characteristics between groups except for RAVi and RA pressure, the latter being invasively measured by right heart catheterization. Notably, patients with increased NT-proBNP at 4-week follow-up had lower NT-proBNP concentrations at baseline.

**Table 5:** Comparison of Baseline Characteristics Between Patients With Decreasing vs Increasing NT-proBNP at 4 Wk

	Decreasing NT-proBNP (n = 100)	Increasing NT-proBNP (n = 110)	*P* Value
Age, y	80 (76.5-83)	80 (76-83)	0.60
BMI, kg/m^2^	25.07 (22.86-29.06)	23.90 (21.97-27.85)	0.12
Female	72 (72)	77 (70)	0.90
EuroSCORE II, %	4.03 (2.44-5.97)	4.52 (2.79-7.67)	0.051
TRI-SCORE, points	4 (3-6)	5 (3.5-6)	0.50
Type of intervention			0.30
TEER	61 (61)	76 (69)	
CardioBand	39 (39)	34 (31)	
Intraprocedural success (TVARC)	71 (71)	78 (71)	>0.90
NYHA functional class			>0.90
I	2 (2)	2 (1.8)	
II	14 (14)	16 (15)	
III	71 (71)	79 (72)	
IV	13 (13)	13 (12)	
HF-hospitalization in the last 12 months	50 (50)	61 (55)	0.50
Hypertension	85 (85)	84 (76)	0.20
Diabetes	18 (18)	30 (27)	0.20
Atrial fibrillation	94 (94)	102 (93)	>0.0.9
CKD	56 (56)	53 (48)	0.30
CAD	32 (32)	46 (42)	0.20
Previous MI	5 (5)	4 (3.6)	0.90
Previous Cardiac Surgery	14 (14)	25 (23)	0.15
CIED	22 (22)	24 (22)	>0.90
Left heart failure phenotypes			0.70
No left heart failure	5 (5)	6 (5.5)	
HFrEF	4 (4)	8 (7.3)	
HFmrEF	11 (11)	15 (14)	
HFpEF	80 (80)	81 (74)	
Loop diuretic agents, mg	30 (17.50-60)	40 (20-60)	0.30
Days of hosp.	5.5 (4-7.5)	6 (5-9)	0.11
Albumin, g/L	42 (40-44)	42 (40-45)	0.30
Hemoglobin, g/dl	12.1 (10.8-13.2)	12.2 (10.8-13.2)	>0.90
eGFR, mL/min/1.73 m^2^	44 (32-54)	41 (33-52)	0.50
NT-proBNP at baseline, ng/L	2,301.5 (1,294-3,325)	1,660.5 (992-2,683)	0.025
Absolute NT-proBNP change, ng/L	−462.5 (−972.5/-175.2)	540.5 (241.8-1300.2)	<0.001
Relative NT-proBNP change, %	−26.1 (−36.2/-14.1)	36.3 (16.8-62.7)	<0.001
NT-proBNP tertile at baseline			0.30
Low	33 (33)	46 (42)	
Intermediate	35 (35)	39 (35)	
High	32 (32)	25 (23)	
LVEF, % (n = 184)	56 (53-62.8)	55 (51-60)	0.20
LAVi, mL/m^2^ (n = 190)	57 (43.3-75)	54.9 (42.768.2)	0.40
PASP, mm Hg (n = 196)	42 (34.3-54.6)	42.5 (35.3-53)	>0.90
RAVi, mL/m^2^ (n = 173)	67.47 (49.84-94.76)	78.05 (59.45-106.44)	0.047
RV basal Diameter, mm (n = 198)	43.39 (40-47.85)	45.8 (41.58-50)	0.078
TAPSE, mm (n = 202)	18 (16-21)	18 (14.5-21)	0.60
FAC, % (n = 185)	36 (31.4-41)	35 (29-40.9)	0.30
TR grade at baseline			0.90
III	60 (60)	65 (59)	
IV	26 (26)	27 (25)	
V	14 (14)	18 (16)	
TR grade at discharge			0.70
No TR	1 (1)	2 (1.8)	
I	35 (35)	35 (32)	
II	39 (39)	38 (35)	
III	18 (18)	29 (26)	
IV	5 (5)	5 (4.5)	
V	2 (2)	1 (0.9)	
TR improvement			>0.90
<2 grades	45 (45)	50 (45)	
≥2 grades	55 (55)	60 (55)	
RA pressure, mm Hg (n = 183) (RHC)	13 (11-17)	15 (12-18)	0.044
PA pressure (mean), mm Hg (n = 149) (RHC)	28.5 (23-32)	28 (24-35)	>0.90
PCWP, mm Hg (n = 138) (RHC)	18 (14-22)	19 (14-23)	0.40
PA saturation, % (n = 140) (RHC)	69.35 (64.9-74)	68.55 (63.45-73.45)	0.20
Cardiac index, l/min/m^2^ (n = 132) (RHC)	2.37 (2-3)	2.34 (2-2.8)	0.60

For each variable, the value in parentheses (n = …) denotes the number of patients with available data. Variables without an “n = …” annotation had complete data with no missing observations.

PA = pulmonary artery; PCWP = pulmonary capillary wedge pressure; RA = right atrium; RHC = right heart catheterization; other abbreviations as in Table 1, Table 2, Table 3 .

**Figure 3:**
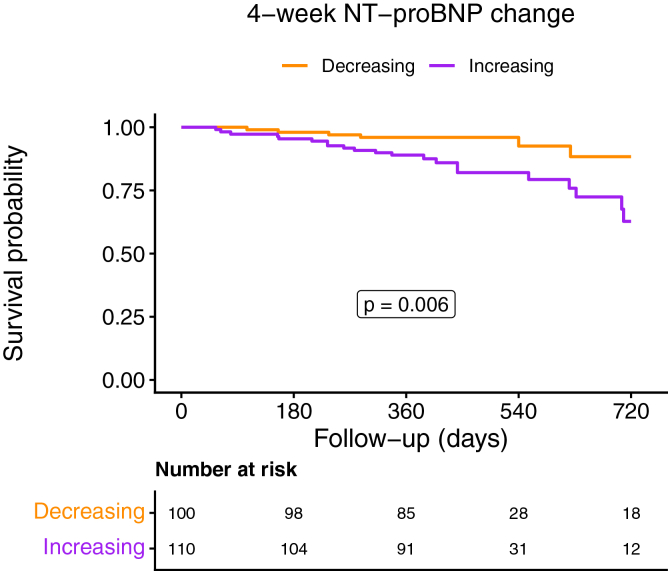
**Kaplan-Meier Curves for All-Cause Mortality According to 4-Week NT-proBNP Trajectory After Transcatheter Tricuspid Valve Intervention** Kaplan-Meier survival curves illustrate all-cause mortality stratified by early NT-proBNP trajectory at 4 weeks after TTVI, categorized as decreasing or increasing NT-proBNP levels. Survival distributions were compared using the log-rank test. Patients with increasing NT-proBNP at early follow-up exhibited significantly worse long-term survival, identifying a high-risk subgroup despite intervention. Abbreviation as in Figure 1.

Kaplan-Meier survival analysis demonstrated a significantly lower all-cause mortality in the group with decreasing NT-proBNP values (log-rank *P* = 0.006) (Figure 3). Subsequently, Cox proportional hazards regression models were performed to explore the prognostic significance of various NT-proBNP concentrations. NT-proBNP level at 4 weeks was strongly associated with all-cause mortality (HR: 7.27; 95% CI: 3.35-15.80; *P* < 0.001), as was the binary change (increasing vs decreasing) in NT-proBNP (HR: 4.02; 95% CI: 1.64-9.87; *P* = 0.002), and the absolute change between baseline and 4 weeks (HR: 1.35 per unit increase; 95% CI: 1.14-1.61; *P* = 0.001). All associations remained statistically significant in multivariable models after adjusting for relevant covariates. Full multivariable regression models are presented in the Supplemental Tables 3, 4 and 5.

## Discussion

To the best of our knowledge, this is the first study to analyze the impact of NT-proBNP concentrations on all-cause mortality in patients with severe TR after TTVI. The baseline characteristics of our cohort were largely comparable to those reported in larger TR referral populations,^12^ supporting the representativeness of our sample.

In this single-center study of 293 patients with severe TR undergoing TTVI, we observed the following:

1 Patients with TR undergoing TTVI show an increased hazard for all-cause mortality with higher baseline NT-proBNP values. This association was independent of relevant clinical and echocardiographic parameters.
2 Baseline NT-proBNP retained its prognostic value even among patients with intraprocedural success, with increasing tertiles remaining associated with worse long-term mortality despite successful intervention.
3 At 4-week follow-up, half of patients had increasing NT-proBNP and the other half decreasing NT-proBNP concentrations.
4 NT-proBNP increase as well as higher absolute NT-proBNP concentrations at early follow-up of 4 weeks after TTVI revealed a high-risk subgroup with higher mortality at 2-year follow-up. The main findings of the study are summarized in the Central Illustration.

**Central Illustration:**
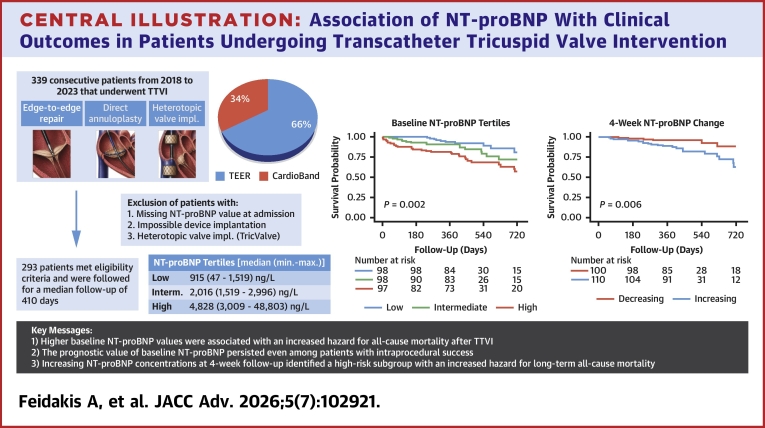
**Association of NT-proBNP With Clinical Outcomes in Patients Undergoing Transcatheter Tricuspid Valve Intervention** In this single-center cohort, 339 consecutive patients undergoing TTVI between 2018 and 2023 were screened, of whom 293 fulfilled eligibility criteria and were included in the final analysis. Patients with tricuspid regurgitation undergoing TTVI showed an increased hazard for all-cause mortality with higher baseline NT-proBNP values, independent of relevant clinical and echocardiographic parameters. This prognostic association persisted even among patients with intraprocedural success. At 4-week follow-up, patients with increasing NT-proBNP concentrations demonstrated significantly worse long-term survival compared with patients showing decreasing values. Early postprocedural NT-proBNP assessment may therefore help identify patients at high-risk for long-term outcomes. NT-proBNP = N-terminal pro-B-type natriuretic peptide; TTVI = transcatheter tricuspid valve intervention; TEER = transcatheter edge-to-edge repair.

NT-proBNP, a universal biomarker of HF, is secreted under pathological conditions primarily by the ventricles and secondarily by the atria, mainly driven by myocardial wall stress.^18^ In the context of severe TR and right HF, increased NT-proBNP reflects myocardial stretch from pressure and/or volume overload,^13^^,^^14^ and is correlated with a dysfunction of the RV and RA.^15^^,^^16^

Furthermore, NT-proBNP can reflect an underlying LV dysfunction, which frequently constitutes the underlying etiology of secondary TR. LV has a greater impact on NT-proBNP levels than RV owing to its substantially larger myocardial mass.^17^ Natriuretic peptides are cleared passively by organs with high rate of blood flow (muscle, liver, and kidney).^18^ As a result, its increase is also illustrative of systemic congestion, occurring through decreased venous return of the aforementioned organs. Accordingly, in the context of TR, NT-proBNP may serve as an indicator of disease progression/staging, LV dysfunction (if present) and venous congestion.

As shown in our analysis, an increased NT-proBNP value at baseline independently predicts long-term outcomes. Evidence from surgical cohorts aligns with this observation: in patients undergoing corrective surgery for isolated severe TR, Yoon et al^19^ demonstrated that higher preoperative NT-proBNP concentrations were associated with significantly increased all-cause mortality and adverse postoperative outcomes, highlighting the relevance of natriuretic peptides for preinterventional risk stratification in patients undergoing surgery for TR.

Importantly, the prognostic value of baseline NT-proBNP persisted even among patients with intraprocedural success. This finding suggests that even after successful TR reduction, patients with elevated baseline NT-proBNP remain at increased risk, likely reflecting more advanced and only partially reversible stages of right-sided HF.

Interestingly, the survival curves of the NT-proBNP tertiles separated predominantly within the first 180 days after TTVI, whereas they remained largely parallel thereafter (Figures 1 and 2). This finding suggests that patients with the highest baseline NT-proBNP values represent a subgroup with advanced right-sided HF, severe myocardial remodeling, and limited hemodynamic reserve. In these patients, the disease process may already have progressed beyond a stage at which mechanical correction of TR alone can substantially alter the natural course of the disease. Consequently, even technically successful intervention may be insufficient to overcome the early hazard associated with advanced right HF.

At the 4-week follow-up, median NT-proBNP remained virtually unchanged (2,016 [1,265-3,833] vs 1,906.5 [1,133.5-3,265.2] ng/L, Wilcoxon signed-rank test, *P* = 0.212). This was not associated with unsuccessful TR reduction and is in concordance with real world data after transcatheter tricuspid valve replacement using the EVOQUE system, presented recently by Angellotti et al^20^ Approximately half of the patients exhibited a decrease in NT-proBNP, whereas the other half showed an increase, irrespective of procedural success. Given that NT-proBNP release reflects myocardial wall stress, this divergent biomarker response suggests heterogeneous hemodynamic adaptations following intervention and warrants consideration of the complex pathophysiological changes induced by TTVI.

Following TTVI, a complex and dynamic hemodynamic rebalancing occurs. The immediate reduction in RA and RV volume/pressure overload alleviates venous congestion;^21^ however, this is accompanied by a sudden increase in effective RV afterload due to the elimination of the low-resistance regurgitant pathway. In parallel, restoration of forward flow across the tricuspid valve leads to an increase in LV preload. This augmented preload, together with the release of pericardial constraint secondary to reduced right-sided chamber volumes, promotes enhanced interventricular interaction and may result in elevated LV filling pressures in specific patients.^22^ As indicated by early NT-proBNP trajectories after TTVI, at least 2 distinct hemodynamic response patterns to TR reduction can be identified: in 1 subgroup, patients exhibit preserved RV systolic reserve, enabling them to tolerate the abrupt increase in effective RV afterload following intervention, along with sufficient LV diastolic compliance to accommodate the resulting rise in filling pressures. In these individuals, relief of RV volume overload leads to reduced RA stretch, improved systemic decongestion, and early reverse remodeling of the right-sided chambers, culminating in a net reduction of global myocardial wall stress, as evidenced by declining NT-proBNP concentrations.

In contrast, a second subgroup demonstrates an adverse hemodynamic response in which elimination of the low-resistance regurgitant pathway unmasks previously compensated RV systolic dysfunction, resulting in an inability to generate adequate forward flow against the increased afterload. In this setting, persistent right-sided wall stress sustains natriuretic peptide release despite successful valve repair. In addition, in some patients, the postinterventional redistribution of myocardial load toward the left ventricle, particularly in the presence of impaired diastolic reserve, may further contribute to rising NT-proBNP levels, reflecting a right-to-left shift in cardiac strain rather than true hemodynamic improvement.

To further explore determinants of the early hemodynamic response to TTVI, baseline clinical, laboratory, and echocardiographic characteristics were compared between responders (defined by a decrease in NT-proBNP within 4 weeks) and nonresponders (defined by an increase in NT-proBNP within 4 weeks). Notably, nonresponders were characterized by larger RA volumes and higher RA pressures at baseline, whereas no relevant differences were observed in parameters assessing RV or LV function, or invasive indices of RV afterload, including pulmonary capillary wedge pressure and mean PA pressure. These findings suggest that structural and hemodynamic alterations at the level of the RA may precede overt RV dysfunction detectable by conventional echocardiographic measures. In addition, in patients with right HF, the RA contributes increasingly to NT-proBNP secretion. Given the thin-walled structure of the right atrium, chronic volume, and pressure overload may represent an early manifestation of impaired RV-RA coupling and predispose to afterload mismatch following tricuspid valve intervention. Furthermore, in patients with long-standing TR and advanced RA remodeling, the capacity for reverse atrial remodeling may be limited, resulting in persistently elevated atrial wall stress and sustained NT-proBNP secretion despite successful reduction of regurgitant volume.

Interestingly, patients with increasing NT-proBNP at 4 weeks had lower baseline NT-proBNP levels. This initially paradoxical finding suggests that baseline NT-proBNP alone may underestimate disease severity in some patients with right-sided HF, as it reflects global myocardial wall stress and is often more strongly influenced by left-sided loading conditions. In our cohort, patients with increasing NT-proBNP showed higher RAVi and higher RA pressure, suggesting more advanced right-sided remodeling and congestion, which may have contributed to subsequent NT-proBNP elevation despite initially lower baseline values and successful intervention.

Both elevated absolute NT-proBNP levels and an increase in NT-proBNP at 4 weeks after TTVI identified subgroups with markedly worse long-term survival. These findings are consistent with prior observations in HF populations. Zile et al^23^ demonstrated that dynamic increases in NT-proBNP during follow-up were strongly associated with the risk of HF hospitalization and cardiovascular death in patients with left-sided HF. Similarly, a recent analysis of Teramoto et al^24^ demonstrated that an increase in NT-proBNP over 6 months was associated with a 27% higher risk of HF hospitalization or all-cause death in HF patients, regardless of HF phenotype or comorbidities.

Beyond HF cohorts, dynamic changes in natriuretic peptide levels after structural valve interventions have also been shown to carry prognostic significance. In the PARTNER trial,^25^ O'Neill et al. demonstrated that patients with AS who exhibited sustained reductions in NT-proBNP after TAVR experienced substantially lower mortality compared with those with persistently elevated or rising NT-proBNP values, underscoring the prognostic utility of serial biomarker assessment in the early postprocedural period. Comparable findings have been reported in the mitral space: Tanaka et al^26^ observed that a >30% reduction in NT-proBNP 2 months after transcatheter mitral valve repair was independently associated with a lower risk of all-cause mortality and HF hospitalization. Taken together, these studies highlight that early postinterventional NT-proBNP trajectories provide important insight into long-term outcomes across different valvular pathologies.

Our study extends these concepts to patients with severe TR undergoing TTVI, highlighting that both persistently elevated and rising NT-proBNP trajectories 4 weeks after the index intervention portend unfavorable long-term prognosis, here reflected by all-cause mortality. In our cohort, patients with increasing NT-proBNP levels had higher RA pressure and larger RAVi at baseline.

Ultimately, early NT-proBNP trajectory does not primarily indicate procedural success of TTVI itself, but rather the persistence of manifold clinically relevant myocardial stress after intervention, which in our cohort may be reflected by more advanced RA disease due to long-standing TR. Consequently, symptomatic improvement, which represents the primary therapeutic goal of TTVI in randomized trials, cannot be reliably inferred from changes in NT-proBNP. In contrast, dynamic NT-proBNP patterns appear to capture the burden of advanced myocardial disease that may persist beyond mechanical correction of TR and therefore provide prognostic information with respect to long-term mortality.

### Limitations

First, this is a retrospective study and as such all relevant limitations inherently associated with retrospective analyses apply here. It is important to note that causal inferences cannot be assumed as the results are hypothesis-generating. In addition, patients with technically unsuccessful implantation were excluded from the final analysis. Although no significant baseline differences were observed compared with the included cohort, this comparison was underpowered because of the small sample size, and residual selection bias cannot be excluded. Second, we included patients with intervention for severe TR which comprised different treatment methods, albeit all being transcatheter treatment methods. Third, we did not include conservatively managed patients with TR and cannot draw conclusions about thresholds for intervention. Furthermore, although Kaplan-Meier curves are shown up to 720 days, the number of patients remaining at risk beyond approximately 18 months was limited; therefore, late survival estimates should be interpreted with caution. Importantly, higher-risk patients according to NT-proBNP levels had similar symptomatic improvement. This finding should not be misinterpreted and translated into discouraging or withholding TR interventions. Future randomized studies with conservative controls are needed to capture the beneficial effect of tricuspid intervention on reducing global myocardial stress and define reliable thresholds for intervention.

## Conclusions

In patients with severe TR undergoing TTVI, both baseline and early postprocedural NT-proBNP levels were strongly associated with long-term all-cause mortality. NT-proBNP provided independent prognostic information beyond established clinical and echocardiographic risk factors. Moreover, an increase in NT-proBNP within the first 4 weeks after intervention identified a subgroup at particularly high risk of adverse outcomes. These findings suggest that NT-proBNP can serve as a valuable biomarker for preinterventional patient selection and early postprocedural surveillance, supporting its integration into the routine management of patients treated with TTVI.

Perspectives **COMPETENCY IN MEDICAL KNOWLEDGE:** Taken together, our results suggest that NT-proBNP assessment before and shortly after TTVI provides complementary information for patient management. Baseline NT-proBNP can potentially serve as a tool for preinterventional patient selection for TTVI. Dynamic changes in NT-proBNP within the first month after intervention may act as an early warning signal identifying patients at high risk for adverse long-term outcomes, prompting closer clinical follow-up and optimization of medical therapy.

**TRANSLATIONAL OUTLOOK:** These findings highlight the central role of neurohormonal activation in the continuum of right-sided HF and underscore the importance of integrating biomarker monitoring into the post-TTVI care pathway.

## Funding support and author disclosures

Dr Iliadis has received travel expenses and consultant honoraria from Abbott and Edwards Lifesciences. All other authors have reported that they have no relationships relevant to the contents of this paper to disclose.
