# Supplementary material for: Association of NT-proBNP With Clinical Outcomes in Patients Undergoing Transcatheter Tricuspid Valve Intervention
Source: JACC Adv. 2026 Jun 17;5(7):102921. doi: 10.1016/j.jacadv.2026.102921 (PMC13311164; doi:10.1016/j.jacadv.2026.102921)
Supplement: Supplemental Material [file mmc1.pdf]

## Supplementary Material

### Alternative stratification thresholds for 4-week NT-proBNP change

NT-proBNP is characterized by substantial biological variability even in clinically stable heart failure patients[1] and is strongly influenced by renal function, with an inverse and increasingly exponential relationship at eGFR values below 90 mL/min/1.73 m².[2] To account for this expected variability, which may partly reflect confounding factors rather than true clinical deterioration or improvement, we additionally examined a three-category stratification strategy based on relative 4-week NT-proBNP change: decrease ≥10%, stable (±10%), and increase ≥10%. However, this categorization did not show a statistically significant association with mortality in our cohort, as illustrated by the Kaplan-Meier analysis in Supplementary Figure 2. This may partly be explained by the unequal distribution of patients across the three groups and the resulting limited statistical power.

We additionally examined whether a ≥30% decrease in NT-proBNP at 4 weeks, a response threshold previously associated with improved outcomes in chronic heart failure populations, could identify a prognostically distinct subgroup after TTVI. In contrast to the findings reported by van Veldhuisen et al., this response criterion did not provide meaningful risk discrimination in our cohort (Supplementary Figure 3). This discrepancy may be explained by important differences between the study populations and timing of assessment. The study by van Veldhuisen et al. primarily included patients with HFrEF in whom NT-proBNP reduction was driven by optimized medical heart failure therapy, whereas the majority of patients in our cohort had preserved LVEF and underwent transcatheter tricuspid intervention, resulting in fundamentally different mechanisms underlying NT-proBNP change. In addition, NT-proBNP change in the prior study was assessed over 48 weeks, while our analysis focused on an early

4-week follow-up period. These differences limit direct comparability and may explain why a ≥30% reduction threshold was not prognostically useful in our cohort.

**Figures**

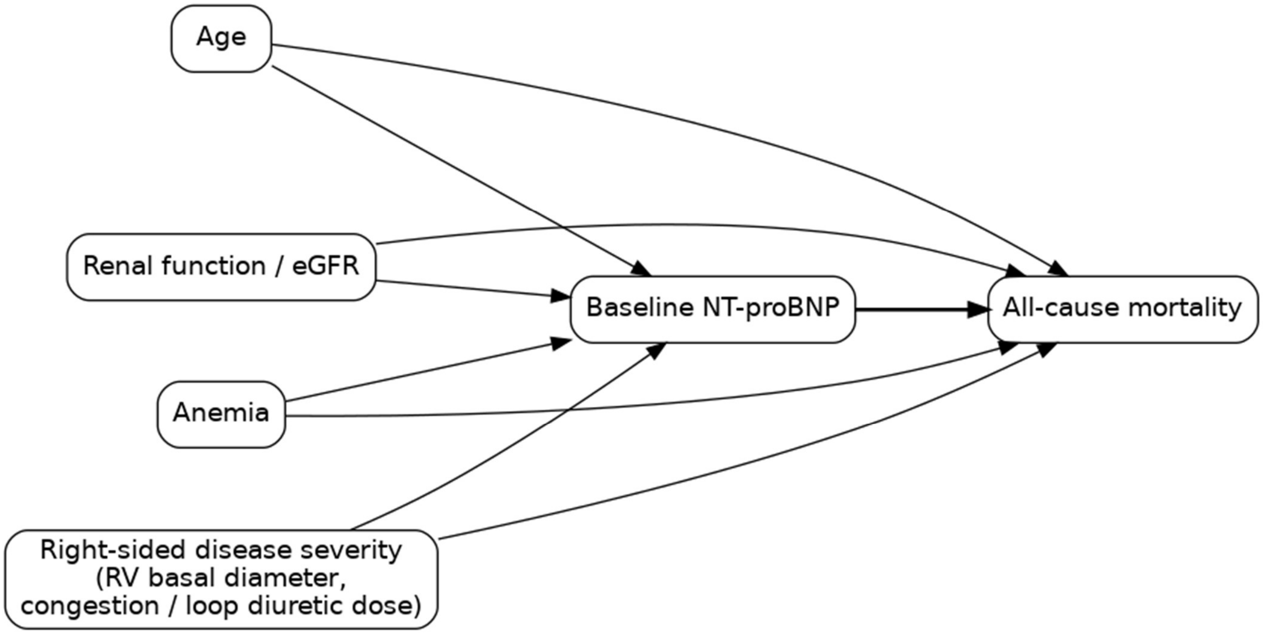


**Supplementary Figure 1**. Directed acyclic graph illustrating the presumed confounding structure between baseline NT-proBNP and all-cause mortality

Caption:

Covariates used for multivariable adjustment were selected according to their potential role as confounders of the association between baseline NT-proBNP and all-cause mortality in patients undergoing TTVI. Arrows indicate presumed directional relationships. RV = right ventricular.

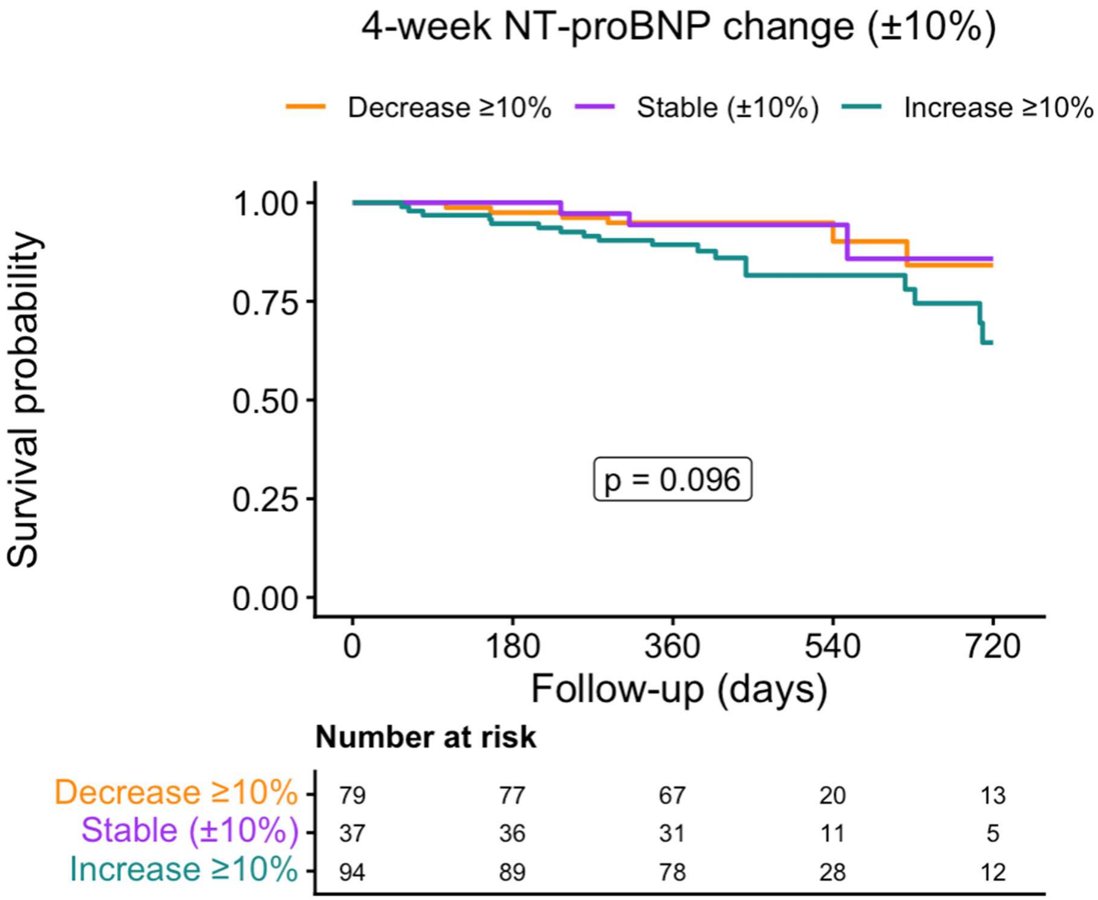


**Supplementary Figure 2**. Kaplan-Meier curves for all-cause mortality according to 10% NT-proBNP change categories at 4-week follow-up after TTVI

<u>Caption</u>:

This figure illustrates all-cause mortality stratified according to relative change in NT-proBNP at 4-week follow-up after TTVI, categorized as decrease ≥10%, stable (±10%), or increase ≥10%. Survival probabilities were estimated using the Kaplan-Meier method and compared using the log-rank test. No significant difference in long-term mortality was observed between groups.

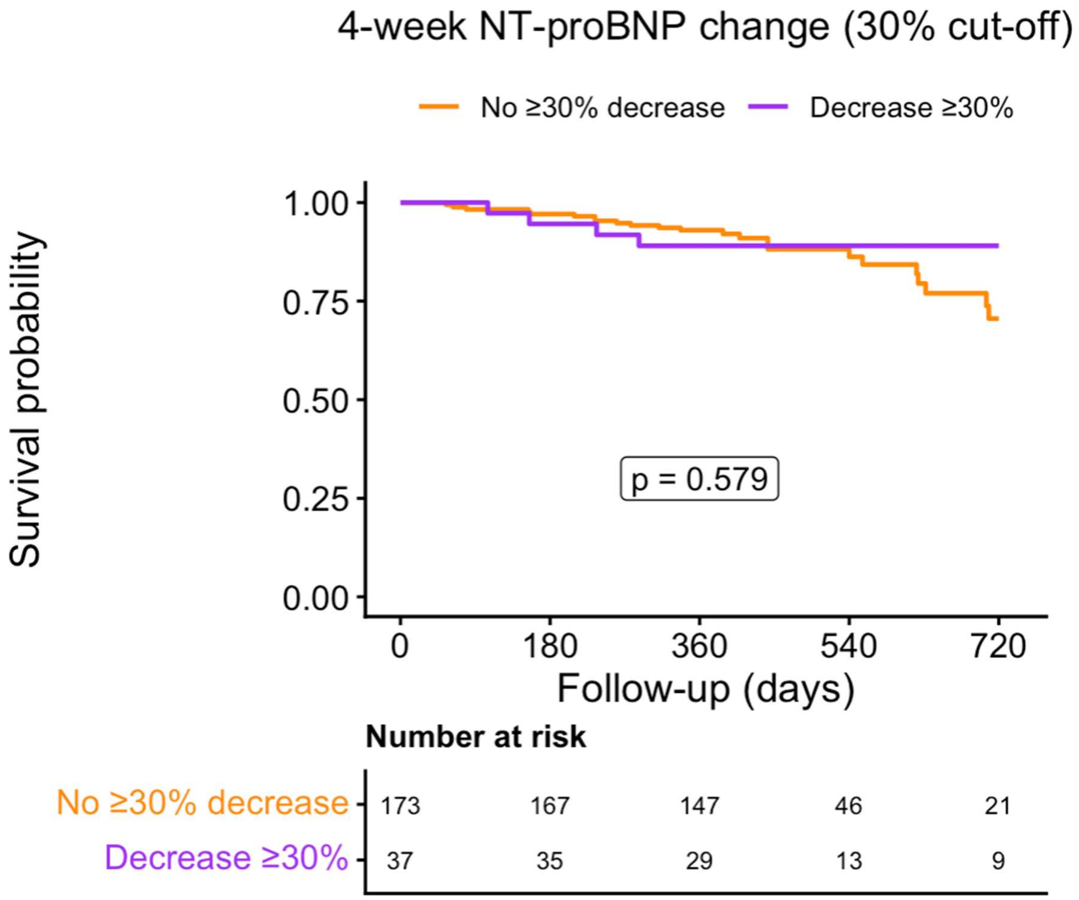


**Supplementary Figure 3**. Kaplan-Meier curves for all-cause mortality according to a ≥30% decrease in NT-proBNP at 4-week follow-up after TTVI

Caption:

This figure illustrates all-cause mortality stratified according to whether patients achieved a ≥30% decrease in NT-proBNP at 4-week follow-up after TTVI. Survival probabilities were estimated using the Kaplan-Meier method and compared using the log-rank test. No significant difference in long-term mortality was observed between patients with and without a ≥30% reduction in NT-proBNP.

## References


1. Täger T, Wiedergruen AK, Fröhlich H, Cebola R, Corletto A, Horsch A, Hess G, Slottje K, Zdunek D, Katus HA, Wians FH Jr, Frankenstein L. Hemodynamic Determinants of the Biologic Variation of N-Terminal Pro-B-Type Natriuretic Peptide in Patients With Stable Systolic Chronic Heart Failure. J Card Fail. 2017 Dec;23(12):835-842. doi: 10.1016/j.cardfail.2017.07.404. Epub 2017 Jul 27. PMID: 28757153.
2. Das SR, Abdullah SM, Leonard D, Drazner MH, Khera A, McGuire DK, de Lemos JA. Association between renal function and circulating levels of natriuretic peptides (from the Dallas Heart Study). Am J Cardiol. 2008 Nov 15;102(10):1394-8. doi: 10.1016/j.amjcard.2008.07.018. Epub 2008 Sep 11. PMID: 18993162.

## Tables

Supplementary Table 1. Baseline characteristics according to technical device implantation success status

| Variable | Fail (N=10) | Success (N=293) | p-value |
|---|---|---|---|
| Age, years | 81 (67-85) | 80 (76-83) | 0.7 |
| Female Sex | 4 (40) | 202 (68.9) | 0.11 |
| BMI, $kg/m^2$ | 27.4 (24.3-32.8) | 24.7 (22.1-28.3) | 0.064 |
| EuroSCORE II, % | 4.8 (2.2-7.7) | 4.4 (2.8-7.2) | 0.8 |
| TRI-SCORE, points | 4.5 (3.5-6.5) | 5 (3-6) | > 0.9 |
| NYHA-Class<br>- Class-I<br>- Class-II<br>- Class-III<br>- Class-IV | <br>0 (0)<br>2 (20)<br>8 (80)<br>0 (0) | <br>4 (1.4)<br>43 (14.7)<br>214 (73)<br>32 (10.9) | 0.7 |
| Diabetes | 0 (0) | 63 (21.5) | > 0.9 |
| Atrial Fibrillation | 8 (80) | 271 (92.5) | 0.4 |
| CAD | 5 (50) | 118 (40.3) | 0.8 |
| CKD | 5 (50) | 150 (51.2) | > 0.9 |
| LVEF, % | 54.5 (45-60) | 55 (52-61.7) | 0.4 |
| TAPSE, mm | 17 (16-17) | 18 (14-21) | 0.6 |
| TR Grade at Baseline | 3 (3-3) | 3 (3-4) | 0.3 |
| Hemoglobin, g/dL | 10.9 (9.9-12) | 12.1 (10.7-13.2) | 0.07 |
| eGFR, $mL/min/1.73m^2$ | 33.5 (25-41) | 41 (31-53) | 0.09 |
| NT-proBNP, ng/L | 3,142 (1,349-5,265) | 2,016 (1,265-3,833) | 0.2 |

Data are presented as median (IQR) for continuous variables and n (%) for categorical variables.

Abbreviations: BMI = Body Mass Index; CAD = Coronary Artery Disease; CKD = Chronic Kidney Disease; eGFR = estimated Glomerular Filtration Rate; LVEF = Left Ventricular Ejection Fraction; NT-proBNP = N-terminal pro-B-type Natriuretic Peptide; NYHA = New York Heart Association; TAPSE = Tricuspid Annular Plane Systolic Excursion; TR = Tricuspid Regurgitation

Supplementary Table 2. Baseline characteristics according to availability of 4-week NT-proBNP measurements among patients completing 4-week follow-up

| **Variable** | **Available (N=210)** | **Missing (N=45)** | **p-value** |
| --- | --- | --- | --- |
| Age, years | 80 (76-83) | 80 (73-84) | 0.6 |
| Female Sex | 148 (70.8) | 28 (60.9) | 0.3 |
| BMI, kg/m$^2$ | 24.2 (22.1-28.9) | 24.9 (22.1-28.3) | 0.9 |
| EuroSCORE II, % | 4.3 (2.7-7.1) | 4.1 (2.8-7.1) | 0.9 |
| TRI-SCORE, points | 5 (3-6) | 5 (3-7) | 0.7 |
| NYHA-Class | | | |
| - Class-I | 4 (1.9) | 0 (0) | |
| - Class-II | 30 (14.4) | 10 (21.7) | |
| - Class-III | 149 (71.3) | 36 (78.3) | 0.06 |
| - Class-IV | 26 (12.4) | 0 (0) | |
| Diabetes | 48 (23) | 6 (13) | 0.2 |
| Atrial Fibrillation | 194 (92.8) | 42 (91.3) | > 0.9 |
| CAD | 78 (37.3) | 23 (50) | 0.2 |
| CKD | 109 (52.2) | 25 (54.3) | > 0.9 |
| LVEF, % | 55 (52-62) | 55.6 (52.1-63.7) | 0.5 |
| TAPSE, mm | 18 (15-21) | 17.5 (14-21) | 0.4 |
| TR Grade at Baseline | 3 (3-4) | 3 (3-4) | 0.5 |
| Hemoglobin, g/dL | 12.1 (10.8-13.2) | 12.3 (10.7-13.5) | 0.4 |
| eGFR, mL/min/1.73m$^2$ | 42 (33-53) | 40 (31-53) | > 0.9 |
| NT-proBNP, ng/L | 1,913 (1,152-3,289) | 2,423 (1,250-4,107) | 0.3 |

Data are presented as median (IQR) for continuous variables and n (%) for categorical variables.

Abbreviations: BMI = Body Mass Index; CAD = Coronary Artery Disease; CKD = Chronic Kidney Disease; eGFR = estimated Glomerular Filtration Rate; LVEF = Left Ventricular Ejection Fraction; NT-proBNP = N-terminal pro-B-type Natriuretic Peptide; NYHA = New York Heart Association; TAPSE = Tricuspid Annular Plane Systolic Excursion; TR = Tricuspid Regurgitation

Supplementary Table 3. Multivariable Cox regression model using NT-proBNP at 4-week follow-up as the primary predictor

| Variable | Univariable Analysis | | | Multivariable Analysis | | |
|---|---|---|---|---|---|---|
| | HR | 95% CI for HR | p-value | HR | 95% CI for HR | p-value |
| $Log_{10}$(NT-proBNP) at 4-week follow-up | 7.271 | 3.347-15.796 | < 0.001 | 10.539 | 3.506-31.679 | < 0.001 |
| Age | 0.986 | 0.955-1.019 | 0.416 | | | |
| Female sex | 0.585 | 0.354-0.967 | 0.037 | 1.436 | 0.620-3.329 | 0.399 |
| Prior MI | 0.740 | 0.232-2.360 | 0.611 | | | |
| Intraprocedural Success (TVARC) | 0.365 | 0.222-0.598 | < 0.001 | 0.753 | 0.331-1.714 | 0.499 |
| Dose of Loop-Diuretics at Baseline (per 20 mg furosemide-equivalent increase) | 1.079 | 1.046-1.113 | < 0.001 | 1.054 | 0.990-1.122 | 0.100 |
| Anemia at Baseline | 1.927 | 1.124-3.302 | 0.017 | 1.330 | 0.516-3.427 | 0.555 |
| eGFR at Baseline | 0.972 | 0.956-0.988 | < 0.001 | 1.000 | 0.970-1.030 | 0.975 |
| LVEDd at Baseline | 1.036 | 0.997-1.077 | 0.071 | | | |
| LVEF at Baseline | 0.980 | 0.955-1.004 | 0.107 | | | |
| RV basal diameter at Baseline | 1.055 | 1.024-1.088 | < 0.001 | 1.086 | 1.032-1.143 | 0.001 |

Anemia was defined according to WHO criteria as hemoglobin <13 g/dL in men and <12 g/dL in women.

HRs for $log_{10}$(NT-proBNP) variables are expressed per 1-unit increase.

Abbreviations: CI = Confidence Interval; eGFR = estimated Glomerular Filtration Rate; HR = Hazard Ratio; LVEDd = Left Ventricular End-Diastolic Diameter; LVEF = Left Ventricular Ejection Fraction; MI = myocardial infarction; NT-proBNP = N-terminal pro-B-type Natriuretic Peptide; RV = Right Ventricle; TVARC = Tricuspid Valve Academic Research Consortium.

Supplementary Table 4. Multivariable Cox regression model using binary NT-proBNP change between baseline and four weeks as the primary predictor

| Variable | Univariable Analysis | | | Multivariable Analysis | | |
|---|---|---|---|---|---|---|
| | HR | 95% CI for HR | p-value | HR | 95% CI for HR | p-value |
| Delta(NT-proBNP) Baseline to 4 Weeks (binary) | 4.024 | 1.640-9.874 | 0.002 | 5.148 | 1.877-14.117 | 0.001 |
| Age | 0.986 | 0.955-1.019 | 0.416 | | | |
| Gender | 0.585 | 0.354-0.967 | 0.037 | 1.067 | 0.479-2.377 | 0.873 |
| Prior MI | 0.740 | 0.232-2.360 | 0.611 | | | |
| Intraprocedural Success (TVARC) | 0.365 | 0.222-0.598 | < 0.001 | 0.542 | 0.241-1.222 | 0.140 |
| Dose of Loop-Diuretics at Baseline (per 20 mg furosemide-equivalent increase) | 1.079 | 1.046-1.113 | < 0.001 | 1.085 | 1.015-1.159 | 0.016 |
| Anemia at Baseline | 1.927 | 1.124-3.302 | 0.017 | 1.482 | 0.615-3.572 | 0.381 |
| eGFR at Baseline | 0.972 | 0.956-0.988 | < 0.001 | 0.974 | 0.949-0.999 | 0.045 |
| LVEDd at Baseline | 1.036 | 0.997-1.077 | 0.071 | | | |
| LVEF at Baseline | 0.980 | 0.955-1.004 | 0.107 | | | |
| Basal Diameter of the RV at Baseline | 1.055 | 1.024-1.088 | < 0.001 | 1.062 | 1.005-1.121 | 0.031 |

Anemia was defined according to WHO criteria as hemoglobin <13 g/dL in men and <12 g/dL in women.

HRs for $log_{10}$(NT-proBNP) variables are expressed per 1-unit increase.

Abbreviations: CI = Confidence Interval; eGFR = estimated Glomerular Filtration Rate; HR = Hazard Ratio; LVEDd = Left Ventricular End-Diastolic Diameter; LVEF = Left Ventricular Ejection Fraction; MI = myocardial infarction; NT-proBNP = N-terminal pro-B-type Natriuretic Peptide; RV = Right Ventricle; TVARC = Tricuspid Valve Academic Research Consortium.

Supplementary Table 5. Multivariable Cox regression model using absolute NT-proBNP change between baseline and 4 weeks as the primary predictor

| **Variable** | **Univariable Analysis** | | | **Multivariable Analysis** | | |
|---|---|---|---|---|---|---|
| | **HR** | **95% CI for HR** | **p-value** | **HR** | **95% CI for HR** | **p-value** |
| Signed log of Absolute Delta(NT-proBNP) Baseline to 4 Weeks | 1.353 | 1.137-1.609 | 0.001 | 1.363 | 1.136-1.635 | 0.001 |
| Age | 0.986 | 0.955-1.019 | 0.416 | | | |
| Gender | 0.585 | 0.354-0.967 | 0.037 | 1.089 | 0.483-2.454 | 0.837 |
| Prior MI | 0.740 | 0.232-2.360 | 0.611 | | | |
| Intraprocedural Success (TVARC) | 0.365 | 0.222-0.598 | < 0.001 | 0.568 | 0.253-1.276 | 0.171 |
| Dose of Loop-Diuretics at Baseline (per 20 mg furosemide-equivalent increase) | 1.079 | 1.046-1.113 | < 0.001 | 1.079 | 1.010-1.153 | 0.025 |
| Anemia at Baseline | 1.927 | 1.124-3.302 | 0.017 | 1.464 | 0.603-3.556 | 0.400 |
| eGFR at Baseline | 0.972 | 0.956-0.988 | < 0.001 | 0.977 | 0.953-1.003 | 0.084 |
| LVEDd at Baseline | 1.036 | 0.997-1.077 | 0.071 | | | |
| LVEF at Baseline | 0.980 | 0.955-1.004 | 0.107 | | | |
| Basal Diameter of the RV at Baseline | 1.055 | 1.024-1.088 | < 0.001 | 1.068 | 1.011-1.128 | 0.019 |

Anemia was defined according to WHO criteria as hemoglobin <13 g/dL in men and <12 g/dL in women.

HRs for $\log_{10}$(NT-proBNP) variables are expressed per 1-unit increase.

Abbreviations: CI = Confidence Interval; eGFR = estimated Glomerular Filtration Rate; HR = Hazard Ratio; LVEDd = Left Ventricular End-Diastolic Diameter; LVEF = Left Ventricular Ejection Fraction; MI = myocardial infarction; NT-proBNP = N-terminal pro-B-type Natriuretic Peptide; RV = Right Ventricle; TVARC = Tricuspid Valve Academic Research Consortium.

STROBE Statement—checklist of items that should be included in reports of observational studies

| | Item No. | Recommendation | Page No. | Relevant text from manuscript |
|---|---|---|---|---|
| **Title and abstract** | 1 | (*a*) Indicate the study's design with a commonly used term in the title or the abstract | 1-2 | We analyzed a cohort of patients with severe tricuspid regurgitation undergoing TTVI. |
| | | (*b*) Provide in the abstract an informative and balanced summary of what was done and what was found | 1-2 | Higher baseline NT-proBNP levels were independently associated with increased long-term all-cause mortality. Early NT-proBNP changes provided additional prognostic information: patients with increasing NT-proBNP levels at 4 weeks showed markedly worse survival, irrespective of procedural success. |
| **Introduction** | | | | |
| Background/rationale | 2 | Explain the scientific background and rationale for the investigation being reported | 4-5 | Despite these advances, identifying reliable prognostic markers in this unique patient population remains a challenge. Established surgical or transcatheter risk scores lack the use of biomarkers. |
| Objectives | 3 | State specific objectives, including any prespecified hypotheses | 4 | The present study aimed to evaluate the prognostic relevance of NT-proBNP levels, both at baseline and four weeks after transcatheter tricuspid valve repair, and to determine their association with long-term mortality in patients with severe, symptomatic TR treated with TTVI. |
| **Methods** | | | | |

| | | | | |
|---|---|---|---|---|
| Study design | 4 | Present key elements of study design early in the paper | 5 | This retrospective observational study included all consecutive patients with symptomatic TR, who underwent percutaneous transcatheter tricuspid valve intervention at our single tertiary care center between January 2018 and December 2023. |
| Setting | 5 | Describe the setting, locations, and relevant dates, including periods of recruitment, exposure, follow-up, and data collection | 5 | This retrospective observational study included all consecutive patients with symptomatic TR, who underwent percutaneous transcatheter tricuspid valve intervention at our single tertiary care center between January 2018 and December 2023. Patients were scheduled for standardized follow-up at four weeks and 12 months after the procedure, which included blood sampling, transthoracic echocardiography (TTE), and clinical examination. |
| Participants | 6 | (*a*) *Cohort study*—Give the eligibility criteria, and the sources and methods of selection of participants. Describe methods of follow-up<br>*Case-control study*—Give the eligibility criteria, and the sources and methods of case ascertainment and control selection. Give the rationale for the choice of cases and controls<br>*Cross-sectional study*—Give the eligibility criteria, and the sources and methods of selection of participants | 5-6 | Patients with unavailable baseline NT-proBNP values or with technically unsuccessful device implantation, defined as failure to successfully deliver and deploy the intended device(s) in tricuspid valve position and retrieve the delivery system, were excluded. Because of their distinct pathophysiological mechanisms and hemodynamic effects, patients who underwent heterotopic caval valve implantation using stent-grafts were also |

| | | | | |
|---|---|---|---|---|
| | | | | excluded. |
| | | (*b*) *Cohort study*—For matched studies, give matching criteria and number of exposed and unexposed<br>*Case-control study*—For matched studies, give matching criteria and the number of controls per case | N/A | Not applicable. |
| Variables | 7 | Clearly define all outcomes, exposures, predictors, potential confounders, and effect modifiers. Give diagnostic criteria, if applicable | 6-9 | The primary endpoint was all-cause mortality during available follow-up. NYHA functional class improvement was assessed as a secondary endpoint and defined as an improvement of at least one NYHA class compared with baseline at 4-week follow-up. Exploratory endpoints included the association of NT-proBNP levels at 4 weeks and absolute change in NT-proBNP between baseline and 4-week follow-up (Δ NT-proBNP) with all-cause mortality. |
| Data sources/ measurement | 8* | For each variable of interest, give sources of data and details of methods of assessment (measurement). Describe comparability of assessment methods if there is more than one group | 6-9 | Clinical, laboratory, and echocardiographic data were obtained retrospectively from the hospital's electronic medical records. Transthoracic echocardiographic assessments were conducted at baseline, discharge, and follow-up visits by experienced sonographers using standardized acquisition protocols in accordance with current ASE/EACVI guidelines. NT-proBNP concentrations were measured using an electrochemiluminescence immunoassay (ECLIA) on the Roche Diagnostics platform. |

| | | | | |
|---|---|---|---|---|
| Bias | 9 | Describe any efforts to address potential sources of bias | 10, 22 | To ensure that exclusion of patients with technically unsuccessful device implantation did not introduce selection bias toward a less advanced disease stage, we compared the baseline characteristics of the excluded patients with those of the final study cohort. First, this is a retrospective study and as such all relevant limitations inherently associated with retrospective analyses apply here. |
| Study size | 10 | Explain how the study size was arrived at | 5, 10 | This retrospective observational study included all consecutive patients with symptomatic TR, who underwent percutaneous transcatheter tricuspid valve intervention at our single tertiary care center between January 2018 and December 2023. A total of 339 patients consecutively treated with TTVI were initially screened. |

Continued on next page

| | | | | |
|---|---|---|---|---|
| Quantitative variables | 11 | Explain how quantitative variables were handled in the analyses. If applicable, describe which groupings were chosen and why | 8-9 | Continuous variables are presented as median (Q1-Q3), while categorical variables were summarized as counts and percentages. NT-proBNP at baseline was analyzed both as a continuous variable and after stratification by tertiles. |
| Statistical methods | 12 | (*a*) Describe all statistical methods, including those used to control for confounding | 7-8 | Group comparisons were performed using the chi-square test for categorical variables, and the Kruskal-Wallis or Mann-Whitney U tests for continuous variables, as appropriate. Univariable and multivariable Cox proportional hazards regression models were employed to assess associations between NT-proBNP variables and all-cause mortality. |
| | | (*b*) Describe any methods used to examine subgroups and interactions | 13 | Device-specific sensitivity analyses demonstrated that baseline logNT-proBNP was significantly associated with all-cause mortality in both TEER patients (HR 2.94, 95% CI 1.44-5.98, p = 0.003) and CardioBand patients (HR 4.10, 95% CI 1.65-10.16, p = 0.002). In an unadjusted interaction Cox model including baseline logNT-proBNP, device type, and their interaction, no significant interaction between device type and baseline NT-proBNP was observed (p for interaction = 0.433), suggesting that the prognostic value of NT-proBNP was not device-specific. |
| | | (*c*) Explain how missing data were addressed | 7 | Missing data were handled on a test-by-test basis (pairwise exclusion). |

| | | | | |
|---|---|---|---|---|
| | | (*d*) *Cohort study*—If applicable, explain how loss to follow-up was addressed<br>*Case-control study*—If applicable, explain how matching of cases and controls was addressed<br>*Cross-sectional study*—If applicable, describe analytical methods taking account of sampling strategy | 6, 14 | Patients were censored at the last available clinical contact if no event had occurred. Seven patients (2.4%) died within the first 30 days following the index procedure, including both in-hospital deaths and those occurring after discharge. while 31 patients (10.6%) were lost to follow-up. |
| | | (*e*) Describe any sensitivity analyses | 13 | Device-specific sensitivity analyses demonstrated that baseline logNT-proBNP was significantly associated with all-cause mortality in both TEER patients (HR 2.94, 95% CI 1.44-5.98, p = 0.003) and CardioBand patients (HR 4.10, 95% CI 1.65-10.16, p = 0.002). |
| **Results** | | | | |
| Participants | 13* | (a) Report numbers of individuals at each stage of study—eg numbers potentially eligible, examined for eligibility, confirmed eligible, included in the study, completing follow-up, and analysed | 10 | A total of 339 patients consecutively treated with TTVI were initially screened. After applying the exclusion criteria, 293 patients were included in the current analysis (16 TricValve patients, 10 technically unsuccessful implantations, and 20 patients with missing baseline NT-proBNP values were excluded). |
| | | (b) Give reasons for non-participation at each stage | 10 | After applying the exclusion criteria, 293 patients were included in the current analysis (16 TricValve patients, 10 technically unsuccessful implantations, and 20 patients with missing baseline NT-proBNP values were excluded). |
| | | (c) Consider use of a flow diagram | N/A | Not applicable. |
| Descriptive data | 14* | (a) Give characteristics of study participants (eg demographic, clinical, social) and information on exposures and potential | 10-13; Tables 1- | Median age was 80 (76-83) years and 69% |

| | | | | |
|---|---|---|---|---|
| | | confounders | 5 | were female. The median NT-proBNP concentration was 2,016 ng/L (1,265-3,833) in the overall cohort. |
| | | (b) Indicate number of participants with missing data for each variable of interest | Tables 2, 3, 5 | For each variable, the value in parentheses (n = …) denotes the number of patients with available data. Variables without an "n = …" annotation had complete data with no missing observations. |
| | | (c) *Cohort study*—Summarise follow-up time (eg, average and total amount) | 12 | During a median follow-up of 410 days (368–593), a total of 63 patients (21.5%) died. |
| Outcome data | 15* | *Cohort study*—Report numbers of outcome events or summary measures over time | 12 | During a median follow-up of 410 days (368–593), a total of 63 patients (21.5%) died. Of these, 33 deaths occurred within the first year, corresponding to 11.3% of the overall cohort and 52.4% of all deaths. |
| | | *Case-control study*—Report numbers in each exposure category, or summary measures of exposure | N/A | Not applicable. |
| | | *Cross-sectional study*—Report numbers of outcome events or summary measures | N/A | Not applicable. |
| Main results | 16 | (*a*) Give unadjusted estimates and, if applicable, confounder-adjusted estimates and their precision (eg, 95% confidence interval). Make clear which confounders were adjusted for and why they were included | 12-13; Table 4 | When assessed as a continuous variable, univariable Cox regression revealed that higher NT-proBNP levels at baseline were strongly associated with an increased mortality risk (HR 3.17, 95% CI 1.82-5.50, $p < 0.001$). This association remained independently significant after multivariable adjustment for clinical and echocardiographic covariates (HR 2.48, 95% CI 1.25-4.91, $p = 0.009$). |
| | | (*b*) Report category boundaries when continuous variables were categorized | 10 | The cut-off values used to define the tertiles were: low tertile: 47-1,519 ng/L, intermediate tertile: 1,519-2,996 ng/L, and |

| | | | |
|---|---|---|---|
| | | | high tertile: 3,009-48,803 ng/L. |
| | (*c*) If relevant, consider translating estimates of relative risk into absolute risk for a meaningful time period | N/A | Not applicable. |

Continued on next page

| | | | | |
|---|---|---|---|---|
| Other analyses | 17 | Report other analyses done—eg analyses of subgroups and interactions, and sensitivity analyses | 13-15 | Baseline NT-proBNP retained its prognostic value even after technically successful TTVI, with its tertiles remaining significantly associated with long-term mortality among patients with intraprocedural success. Kaplan-Meier survival analysis demonstrated a significantly lower all-cause mortality in the group with decreasing NT-proBNP values (log-rank p = 0.006; Figure 3). |
| **Discussion** | | | | |
| Key results | 18 | Summarise key results with reference to study objectives | 16 | In this single-center study of 293 patients with severe TR undergoing TTVI, we observed the following: |
| Limitations | 19 | Discuss limitations of the study, taking into account sources of potential bias or imprecision. Discuss both direction and magnitude of any potential bias | 22 | First, this is a retrospective study and as such all relevant limitations inherently associated with retrospective analyses apply here. It is important to note that causal inferences cannot be assumed as the results are hypothesis-generating. |
| Interpretation | 20 | Give a cautious overall interpretation of results considering objectives, limitations, multiplicity of analyses, results from similar studies, and other relevant evidence | 16-22 | Ultimately, the early NT-proBNP trajectory does not primarily indicate procedural success of TTVI itself, but rather the persistence of manifold clinically relevant myocardial stress after intervention, which in our cohort may be reflected by more advanced RA disease due to long-standing TR. |
| Generalisability | 21 | Discuss the generalisability (external validity) of the study results | 16, 22-23 | The baseline characteristics of our cohort were largely comparable to those reported in larger TR referral populations, supporting the representativeness of our sample. Future randomized studies with conservative controls |

| | | | | |
|---|---|---|---|---|
| | | | | are needed to capture the beneficial effect of tricuspid intervention on reducing global myocardial stress and define reliable thresholds for intervention. |
| **Other information** | | | | |
| Funding | 22 | Give the source of funding and the role of the funders for the present study and, if applicable, for the original study on which the present article is based | 1 | Funding: none |

*Give information separately for cases and controls in case-control studies and, if applicable, for exposed and unexposed groups in cohort and cross-sectional studies.

**Note:** An Explanation and Elaboration article discusses each checklist item and gives methodological background and published examples of transparent reporting. The STROBE checklist is best used in conjunction with this article (freely available on the Web sites of PLoS Medicine at http://www.plosmedicine.org/, Annals of Internal Medicine at http://www.annals.org/, and Epidemiology at http://www.epidem.com/). Information on the STROBE Initiative is available at www.strobe-statement.org.
